# CHALLENGE and Face Your Fears: Virtual Reality Treatment for Auditory Hallucinations and Paranoid Ideations

**DOI:** 10.1192/j.eurpsy.2022.82

**Published:** 2022-09-01

**Authors:** L. Glenthøj, L. Smith, L. Mariegaard, A.S. Due, A. Christensen, M. Christensen, D. Vernal, U. Jeppesen, M. Nordentoft

**Affiliations:** 1 Mental Health Center Copenhagen, Core-copenhagen Research Center For Mental Health, Hellerup, Denmark; 2 Mental Health Centre in the Southern part of Denmark, Esbjerg Department, Esbjerg N, Denmark; 3 Aalborg University Hospital, Outpatient Unit For Schizophrenia, Aalborg, Denmark; 4 CORE-Copenhagen Research Center for Mental Health, Mental Health Center Copenhagen, Hellerup, Denmark

**Keywords:** auditory hallucinations, schizophrénia, Psychotherapy, virtual reality

## Abstract

Background: Many patients suffering from schizophrenia spectrum disorders continue having distressing auditory hallucinations and paranoid ideations despite receiving current treatment. Virtual reality assisted treatment offers the potential of advancing current psychotherapies for psychotic symptoms by creating virtual environments that can elicit responses (e.g. thoughts, feelings, behaviours) mirroring real-world settings. In two large-scale randomised clinical trials, we are investigating whether targeted virtual reality assisted psychotherapy can reduce psychotic symptoms and increase daily life functioning and quality of life. The CHALLENGE trial examines whether nine sessions of virtual reality-assisted psychotherapy is superior to nine sessions of standard treatments in reducing the severity, frequency, and distress of auditory hallucinations in patients with psychosis. In the Face your Fears trial we are investigating whether virtual reality assisted cognitive behavioral therapy (CBT) is superior to standard CBT in reducing levels of paranoid ideation in patients with psychosis spectrum disorders. Methods: The CHALLENGE and Face your Fears trials are randomised, assessor-blinded parallel-groups superiority clinical trials, allocating a total of 266 and 256 patients, respectively to either the experimental intervention or a control condition. The trials are currently enrolling patients; thus, no quantitative data is available yet. The main objective of this presentation is to give a qualitative account of this new psychotherapeutic methods as it is applied in both trials. Results: Qualitative data comprising case descriptions and video material will be presented at the conference. Discussion: The preliminary findings indicate great potential for these innovative treatments albeit important concerns regarding implementation will be raised.

**Disclosure:**

No significant relationships.

